# Calcium Chelidonate: Semi-Synthesis, Crystallography, and Osteoinductive Activity In Vitro and In Vivo

**DOI:** 10.3390/ph14060579

**Published:** 2021-06-17

**Authors:** Elena Avdeeva, Ekaterina Porokhova, Igor Khlusov, Tatyana Rybalova, Elvira Shults, Larisa Litvinova, Valeria Shupletsova, Olga Khaziakhmatova, Irina Sukhodolo, Mikhail Belousov

**Affiliations:** 1 Department of Pharmaceutical Analysis, Siberian State Medical University, 634050 Tomsk, Russia; rybalova@nioch.nsc.ru; 2 Department of Morphology and General Pathology, Siberian State Medical University, 634050 Tomsk, Russia; porohova_e@mail.ru (E.P.); khlusov63@mail.ru (I.K.); staranie@mail.ru (I.S.); 3 Research School of Chemistry & Applied Biomedical Sciences, Tomsk Polytechnic University, 634050 Tomsk, Russia; 4 Center of Spectral Investigations, Novosibirsk Institute of Organic Chemistry, Siberian Branch, 630090 Novosibirsk, Russia; mvb63@mail.ru; 5 Laboratory of Medicinal Chemistry, Novosibirsk Institute of Organic Chemistry, Siberian Branch, 630090 Novosibirsk, Russia; schultz@nioch.nsc.ru; 6 Center for Immunology and Cell Biotechnology, Immanuel Kant Baltic Federal University, 236041 Kaliningrad, Russia; larisalitvinova@yandex.ru (L.L.); vshupletsova@mail.ru (V.S.); hazik36@mail.ru (O.K.)

**Keywords:** calcium chelidonate, semi-synthesis, X-ray diffraction analysis, osteogenic activity

## Abstract

Calcium chelidonate [Ca(ChA)(H_2_O)_3_]_n_ was obtained by semi-synthesis using natural chelidonic acid. The structure of the molecular complex was determined by X-ray diffraction analysis. The asymmetric unit of [Ca(ChA)(H_2_O)_3_]_n_ includes chelidonic acid coordinated through three oxygen atoms, and three water ligands. The oxygen atoms of acid and oxygen atoms of water from each asymmetric unit are also coordinated to the calcium of another one, forming an infinite linear complex. Calcium geometry is close to the trigonal dodecahedron (D2d). The intra-complex hydrogen bonds additionally stabilize the linear species, which are parallel to the axis. In turn the linear species are packed into the 3D structure through mutual intercomplex hydrogen bonds. The osteogenic activity of the semi-synthetic CaChA was studied in vitro on 21-day hAMMSC culture and in vivo in mice using ectopic (subcutaneous) implantation of CaP-coated Ti plates saturated in vitro with syngeneic bone marrow. The enhanced extracellular matrix ECM mineralization in vitro and ectopic bone tissue formation in situ occurred while a water solution of calcium chelidonate at a dose of 10 mg/kg was used. The test substance promotes human adipose-derived multipotent mesenchymal stromal/stem cells (hAMMSCs), as well as mouse MSCs to differentiate into osteoblasts in vitro and in vivo, respectively. Calcium chelidonate is non-toxic and can stimulate osteoinductive processes.

## 1. Introduction

The use of substances with osteoprotective properties is relevant for treating several diseases associated with a defect of bone or violation of bone metabolism [1,2,3,4,5,6,7]. The search for delivery systems and bone targeting of therapeutic agents is a significant problem [5,7,8,9]. Osteoprotective trace elements (calcium, magnesium, strontium, phosphate) significantly impact the processes of bone regeneration and the normal bone structure [5,10,11,12,13,14,15]. At the same time, natural organic biomolecules (organic acids, flavonoids, polysaccharides) can bind minerals, improving their bioavailability and at the same time are themselves osteogenic agents [2,16,17,18,19]. Organic molecules with chelating properties can deliver mineral components to tissues and lead to an increase in the selectivity of their therapeutic effect on bone tissue [5]. Thus, high osteoprotective activity was revealed in chelated compounds of calcium and magnesium with organic acids [19].

There are many metal-binding biomolecules, including in plant objects [20,21,22]. Some plants can accumulate metal ions in their shoots at significantly higher concentrations than other plants, while not having a physiological need for such a high concentration [23]. It is revealed that plants organic acids of have a significant role in the hyperaccumulation of metals [21,24,25]. One of these compounds is natural chelidonic acid, found in several plants [26,27,28]. Chelidonic acid is a ligand of organometallic compounds of natural and synthetic origin [29,30,31].

It was revealed that *Saussurea controversa* DC (Asteraceae) contains a high concentration of chelidonic acid and can be a source for its isolation [32]. *S. controversa* also contains calcium chelidonate, which showed high osteogenic activity in vitro and enhanced osteogenic differentiation of mesenchymal stromal cells [32]. The ethanol extract obtained from the S. *controversa* leaves and its fraction containing calcium chelidonate showed osteogenic activity in vivo in experimental osteomyelitis in rats [33,34]. Thus, natural calcium chelidonate is promising for engineering as an osteoprotective drug. Simultaneously, the content of calcium chelidonate differs in the samples of raw materials and most likely its biosynthesis depends on the content of calcium in the soil. For pharmaceutical purposes, it will be advisable to obtain calcium chelidonate by a semi-synthetic method. The calcium chelidonate (CaChA) obtained by the semi-synthetic method has an identical structure [Ca(ChA)(H_2_O)_3_]_n_ with the natural analog previously isolated from the extract of *Saussurea controversa* leaves, was confirmed by X-ray analysis and showed pronounced osteoinductive activity in vitro and in vivo. 

## 2. Results

### 2.1. Crystallography of Calcium Chelidonate ([Ca(ChA)(H_2_O)_3_]_n_)

**Figure 1:**
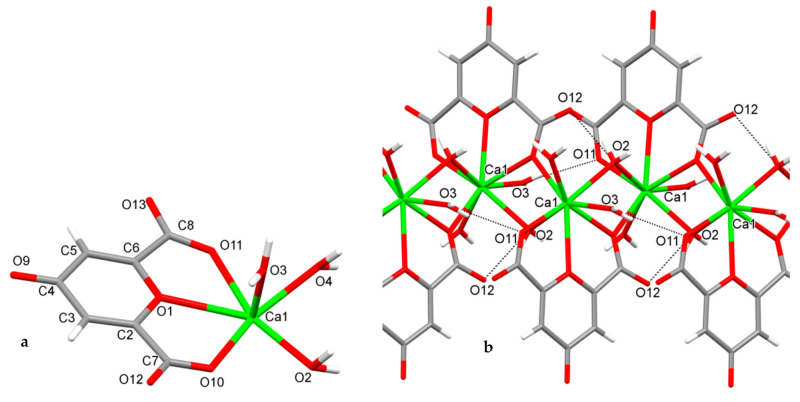
The asymmetric unit (**a**) and the fragment (**b**) of infinite 8-coordinated calcium complex [Ca(ChA)(H_2_O)_3_]_n_ with intracomplex H-bonds (O3–H…O11 and O2–H…O12).

The structure of the eight-coordinated calcium complex [Ca(ChA)(H_2_O)_3_]n was determined by the single crystal X-ray analysis (Figure 1). Crystallographic data for semi-synthetic [Ca(ChA)(H_2_O)_3_]_n_: orthorhombic, *Pna2*(1), a 8.380(2), b 19.702(4), c 6.1653(14) Å, *V* 1017.9(4) Å^3^, *Z* 4, *D*_calcd_ 1.802 g·cm^−3^, *μ*(Mo-*K*α) 0.655 mm^−1^, F(000) 568, (θ 2.64–25.93°, completeness 99.1%), T 296(2) K, colorless, (0.30 × 0.09 × 0.005) mm^3^, transmission 0.7714–0.8620, 9349 measured, 1966 independent (*R*_int_ 0.0487), 179 parameters, 7 restraints, *R*_1_ 0.0347 (for 1736 observed *I >* 2*σ*(*I*)), *wR*_2_ = 0.0801 (all data), GOOF 1.065, largest diff. peak and hole 0.322 e.A^−3^ and −0.220 e.A^−3^ (Table S1).

The reflection intensity was corrected for absorption using the SADABS program [35]. The structure of the sample was determined using the program SHELXS-97 [36] and refined by the anisotropic (isotropic for H atoms) method of least squares of all reflections in accordance with SHELX-97 [36].

The positions of the hydrogens of C3 and C5 were calculated geometrically and refined in the riding model. All water hydrogens’ positions in semi-synthetic crystals were taken from a different map and refined with the restriction of O–H bond length 0.85 Å. Crystallographic data of semisynthetic calcium chelidonate (supplementary publication no. CCDC 2075308) have been deposited at the Cambridge Crystallographic Data Center (deposit@ccdc.cam.ac.uk; www.ccdc.cam.ac.uk (accessed on 5 April 2021)).

The asymmetric unit of [Ca(ChA)(H_2_O)_3_]_n_ includes chelidonic acid coordinated through O1, O10, O11, and three water ligands (Figure 1a). The atoms O10 of acid and O2 of water from each asymmetric unit are also coordinated to the calcium of another one, forming an infinite linear complex (Figure 1b). Calcium geometry is close to the trigonal dodecahedron (D2d); its geometrical parameters are given in Table 1. 

The intra-complex hydrogen bonds O3–H3A…O11 and O2–H2A…O12 (Table 2) additionally stabilize the linear species, which are parallel to axis *c*. In turn the linear species are packed into the 3D structure through mutual intercomplex hydrogen bonds (Table 2).

### 2.2. In Vitro and In Vivo Study of the Osteoinductive Activity of Calcium Chelidonate

**Figure 2:**
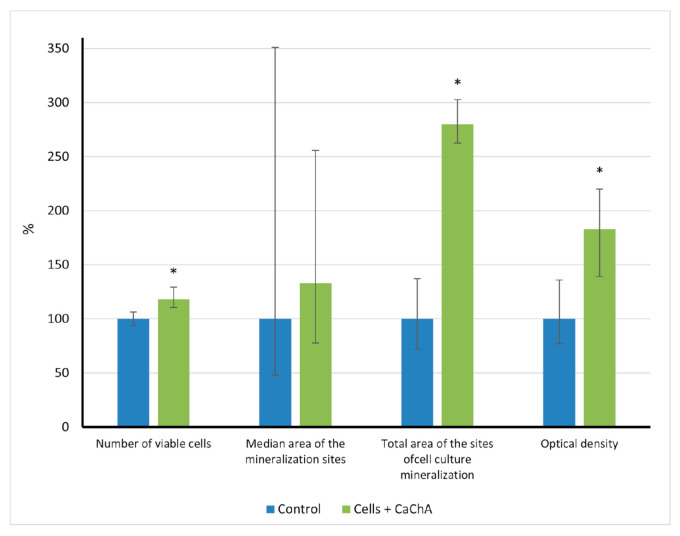
The in vitro indices (% of control) of human adipose-derived multipotent mesenchymal stromal/stem cells after 21 days of culture in the presence of water solvent (control) or 10 mg/L of CaChA in water solution, Me(Q1–Q3). * Statistical differences (*p* < 0.05) with the control according to a Mann–Whitney test.

**Figure 3:**
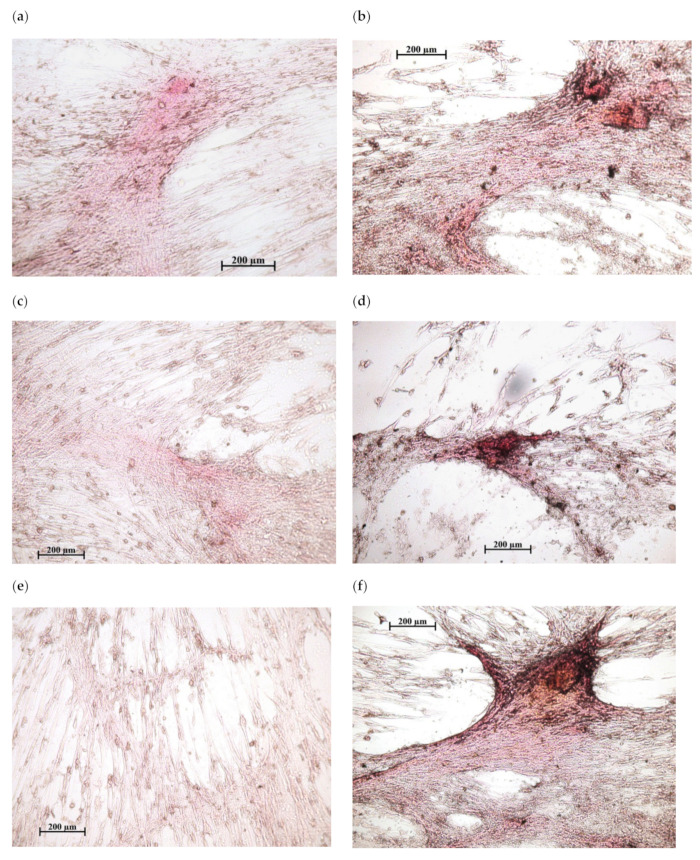
In vitro osteogenic differentiation of human adipose-derived multipotent mesenchymal stromal/stem cells after 21 days of culture in a standard nutrient medium: (**a**,**c**,**e**)—variants of poor diffuse coloring in control culture (addition of water solvent); (**b**,**d**,**f**)—mineralization nodules after the addition of 10 mg/L of CaChA in water solution. Staining with alizarin red S. Magnification ×200. Scale bars 200 µm.

In vitro results showed (Figure 2) that calcium chelidonate(CaChA) at a dose of 10 mg/L statistically increased the number of viable hAMMSCs compared with control culture after 21-day cultivation in a standard nutrient medium. No significant elevation of an average area of the individual sites of mineralization of the extracellular matrix (ECM) was observed. However, increased total area (>250% of the control) and optical density (almost twice as much compared to the control) of ECM mineralization sites were identified by alizarin red S staining (Figure 3). 

**Figure 4:**
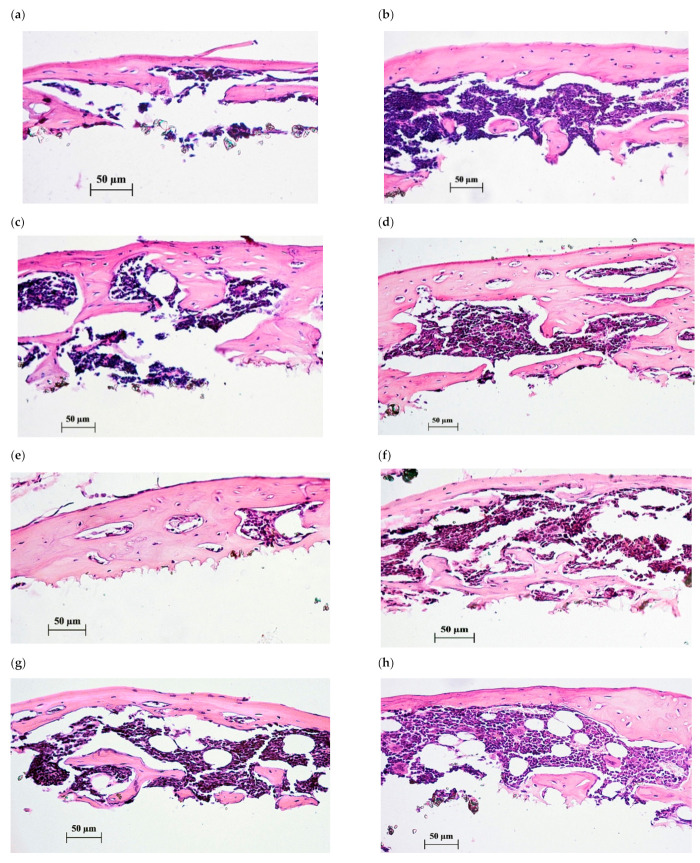
Histological sections of tissue lamellae grown on CaP-coated Ti substrates after 45 days of subcutaneous test in mice. Bone tissues with marrow are shown after 35-day peroral administration of water solvent (**a**–**c**) or 10 mg/L of CaChA in water solution (**d**–**h**). Hematoxylin–eosin staining. Magnification ×200. Scale bars 50 µm.

In vivo macrovisual observation of the subcutaneous sites surrounding the Ti implants with CaP coating showed no signs of inflammation, hypersensitivity, or tissue sensation in all test groups. A thin desmogenous capsule surrounded samples and was easily removed. Tissue lamellae formed by bone marrow were inspected on the microarc CaP surfaces, and the incidence of tissue lamella formation was 80% (four out of five samples in the control, Table 3). The incidence of the control bone tissue outcome in the composition of tissue lamellae achieved 75 % (three out of four samples, Table 3, Figure 4). One tissue lamella with vascularized loose irregular connective tissue on the surface of the CaP-coated Ti samples implanted in control mice can be classified as a failure of implantation. In turn, the incidences of tissue growth and bone formation in lamellae in situ were 100% (five out of five cases) in the case of in vivo CaChA administration (Table 3, Figure 4).

The areas of tissue lamellae increased up to 309–915% compared with the corresponding initial areas of bone marrow columns (Table 3). For all this, 2.5-fold statistical elevation of tissue lamella areas (from 23.65 mm^2^ to 57.4 mm^2^) caused by CaChA was observed. Highly likely, this increase suggests the benefit of the CaChA on promoting bone marrow adhesion, motility and proliferation on the surface of CaP coating under constant biomechanical cyclic loads caused by movement of muscles and the skin of mice.

Histological evaluation of lamella cross sections (Figure 4) showed a formation of membrane reticulated and trabecular bones with lacunae saturated by red marrow to varying degrees. No statistical differences in the cross section features of tissue lamellae were detected for the groups tested with the help of histomorphometric analysis (Table 3). 

## 3. Discussion

When using osteoprotective minerals as medicines for bone regeneration [5,10,11,12,13,14,15], the search for delivery systems and bone targeting of therapeutic agents is a significant problem [5,7,8,9]. Organic molecules with chelating properties can be a way of delivering mineral components to tissues, increasing their bioavailability, and leading to an increase in the selectivity of their therapeutic effect on bone tissue [5]. Such delivery systems can be natural biologically active substances such as organic acids, flavonoids, and polysaccharides [2,16,17,18,19]. One of these compounds is natural chelidonic acid, a ligand of organometallic compounds of natural and synthetic origin [29,30,31].

X-ray diffraction analysis showed that the structure of the semisynthetic calcium chelidonate and the sample isolated from natural raw materials is absolutely identical [32]. In in vitro experiments, natural calcium chelidonate (CaChA) showed enhanced osteogenic differentiation of mesenchymal stem/stromal cells [32]. The fraction from S. *controversa* extract containing CaChA showed osteogenic activity in vivo in experimental osteomyelitis in rats [33,34]. At the same time, it is advisable to obtain CaChA by a semi-synthetic method for pharmaceutical purposes because its content in the samples of raw materials is not constant and the yield of the final product is small. CaChA in crystalline form was obtained by a semi-synthetic method using natural chelidonic acid and anhydrous calcium chloride with a yield of up to 82%. The osteogenic activity of the obtained semi-synthetic CaChA was studied in vitro on 21-day hAMMSC culture and in vivo in mice using ectopic (subcutaneous) implantation CaP-coated Ti plates saturated with syngeneic bone marrow.

Osteoinduction (OI) means that primitive, undifferentiated stem cells are stimulated to develop into bone-forming cells (osteoblasts) [37] and to promote ectopic bone formation in vivo [38]. Suppose OI occurs through the transitional stage of cartilage (so-termed endochondral ossification), the bone marrow forms [39]. Ectopic bone regeneration is triggered by activated MSCs that differentiate into osteoblasts when they are in close contact with an osteogenic material [40].

Most likely, the CaChA could not penetrate the ECM and cause direct calcification of cell culture because the individual sites of alizarin red S staining were equal with the control values. The substance at a dose of 10 mg/L contributes to the survival of hAMMSCs and stimulates stem cells to differentiate into osteoblasts mainly.

We had received similar results previously [32]. Therefore, CaChA possesses OI features in vitro according to the definition of Albrektsson et al. (2001) [37].

CaP materials induce ectopic bone formation when implanted under the skin or in muscle [41]. The incidence of subcutaneous ectopic osteogenesis (SEO) usually varied from 67% to 100%, while it is triggered by syngeneic bone marrow with micro-arc CaP coatings on Ti substrates [42]. The used variant of EO simulates the remodeling of the bone/marrow system in situ. Ectopically implanted bone marrow undergoes a regenerative process that recapitulates marrow ontogeny; this process is possible because the marrow tissue has the considerable angiogenic potential [43] and is a source of mesenchymal stem/stromal cells (MSCs) [44].

The received data (Table 3) correspond to our previous results. Therefore, an artificial CaP surface is a scaffold for de novo bioengineering of MSC microenvironment necessary for the regeneration of the bone/bone marrow system. After that, the stability of SEO caused by the samples with micro-arc CaP allowed investigation of the osteogenic effect of CaChA introduced per os on mouse MSC differentiation into osteoblasts in situ.

Since the initial areas of bone marrow columns placed in vitro over the CaP coatings did not differ statistically (Table 3), the difference in the areas of the tissue lamellae and the type of tissue formed on the CaP coating surface were mainly influenced by the CaChA administration. The substance caused some increase in the incidence of the lamella formation, and 2.5-fold augmentation of the tissue lamellae areas compared with that in control mice (administration of water solvent only). Histological assay of the tissue lamellae grown on the surface of CaP coatings demonstrated a formation of bone tissue filled with red bone marrow in 100% of cases versus 75% in the control (Figure 4, Table 3). 

There were no statistical differences between the areas of newborn bone per individual cross section according to histomorphometric analysis (Table 3). It is similar to in vitro results when the median areas of individual ECM mineralization sites did not differ, too (Figure 2). Simultaneously, an increase in the area of tissue lamellae and the percentage of bone formation in tissue lamellae and cross sections (Table 3) suggests the benefit of the CaChA in vivo administration for stability of SEO values in situ. Similar to the in vitro effect of CaChA (Figure 2), the test substance seems to promote MSC viability, adhesion, and efficacy of their osteogenic differentiation (e.g., number of MSCs differentiated into osteoblasts) on the CaP surface of implants under biomechanical cyclic loads caused by the movement of mouse muscles and skin. Therefore, the OI activity of CaChA can be concluded in vivo according to [38] point of view. 

The mechanisms of the OI effect of CaChA on MSCs will be under further consideration. At least, as we proposed earlier [32], small molecules of Ca-containing CaChA may influence similarly to calcium (in part, through Ca^2+^–sensing receptor) [45] and/or osteogenic small molecules such as β-glycerophosphate, dexamethasone, and ascorbic acid, as well adenosine (via phosphate–adenosine triphosphate (ATP)–adenosine A2b receptor (A2bR) axis) and helioxanthin derivative 4-(4-methoxyphenyl)-pyrido[4 0.30:4.5]thieno-[2,3-b]pyridine-2-carboxamide [46].

## 4. Materials and Methods

### 4.1. General Experimental Procedures

UV spectra were obtained on an HP 8453 UV-Vis spectrometer (Hewlett-Packard, Germany) in EtOH solutions (10^−4^ mol/L). IR spectra were obtained on a Nicolet 5700 (FT-IR, Thermo Fisher Scientific, Waltham MA, USA) in tablets with potassium bromide. The inorganic components were studied by inductively coupled plasma mass spectrometry using Agilent 7900 JP 14080159 (Agilent Technologies, Tokyo, Japan) using the Speedwave MWS TM-3+ microwave system for organic matrix decomposition. For X-ray diffraction analysis, a Bruker KAPPA APEX II diffractometer (Bruker AXS, Karlsruhe, Germany) equipped with a two-dimensional CCD detector (MoKα radiation with a graphite monochromator, ω-φ scanning) was used.

### 4.2. Semi-Synthesis of Calcium Chelidonate

For the semi-synthesis of calcium chelidonate, chelidonic acid was used, obtained from natural raw materials, as described earlier [32]. The structure of the obtained sample of chelidonic acid was proved by NMR and MS/MS analysis, the purity of the sample was established by HPLC (the content of chelidonic acid in the sample is not less than 99.9%). An aqueous solution of 10% sodium hydroxide was added to an aqueous solution of chelidonic acid (0.101 g, 0.5 mmol; in 10 mL H_2_O) to a pH of 7–8. The resulting solution was slowly added to an aqueous calcium chloride anhydrous solution (CaCl_2_ (Atotech, Berlin, Germany); 0.111 g, 1 mmol; in 5 mL H_2_O). The resulting mixture was left to form a precipitate (20–25 °C, 2 h). Then, the precipitate was filtered, washed with small portions of cold water until a negative reaction with the chloride ion, and recrystallized from water (yield 80–82%).

### 4.3. Characterization of Calcium Chelidonate ([Ca(ChA)(H_2_O)_3_]n)

White crystalline powder (H_2_O); calcium content 0.145 g/g; UV (EtOH)nm: 274; IR (KBr, *ν*, cm^−1^): 3516, 3273, 3073, 2827, 1639, 1617, 1597, 1410, 1357, 1315, 1134, 1122, 973, 956, 921, 906, 806, 744, 723, 623, 548, 465; Anal. calc. for C_7_H_8_O_9_Ca(276.15): C 30.43, H 2.90, Ca 14.49; found: C 31.49, H 2.93, Ca 14.50.

Crystallographic data: C_7_H_8_CaO_9_ *M* 276.21, Orthorhombic, *Pna2(1)*, a 8.380(2), b 19.702(4), c 6.1653(14) Å, *V* 1017.9(4) Å^3^, *Z* 4, *D*_calcd_ 1.802 g·cm^−3^, *μ*(Mo-*K*α) 0.655 mm^−1^, F(000) 568, (θ 2.64–25.93°, completeness 99.1%), T 296(2) K, colorless, (0.30 × 0.09 × 0.005) mm^3^, transmission 0.7714–0.8620, 9349 measured, 1966 independent (*R*_int_ 0.0487), 179 parameters, 7 restraints, *R*_1_ 0.0347 (for 1736 observed *I >* 2*σ*(*I*)), *wR*_2_ = 0.0801 (all data), GOOF 1.065, largest diff. peak and hole 0.322 e.A^−3^ and –0.220 e.A^−3^.

### 4.4. Cell Isolation

Lipoaspirate was isolated from a healthy male volunteer who was undergoing liposuction for aesthetic reasons in the surgery hospital. Informed consent of volunteers for the procedure and an approval of the Local Ethics Committee of Innovation Park, Immanuel Kant Baltic Federal University (Kaliningrad, Russia; permission no. 1 on 28 February 2019) were obtained. A stromal vascular fraction (SVF) and processed lipoaspirate (PLA) were isolated according to Zuk et al. (2001) [47] as described previously [48]. To define the multipotent origin of viable fibroblast-like adherent PLA cells, the minimal criteria of the International Society for Cellular Therapy (ISCT) and the International Federation for Adipose Therapeutics and Science (IFATS) [49,50] were used. Cell immunophenotype (CD105, CD73, and CD90 positive markers (>90%) and lack (<2%) expression of CD45, CD34, CD20, or CD14) were detected. Multilineage differentiation of cells into osteoblasts, adipocytes and chondrocytes with reagent using a StemPro^®^ Differentiation Kit (Thermo Fisher Scientific, Waltham, MA, USA) as well differential staining by alizarin red S (Sigma-Aldrich, St. Louis, MO, USA), alcian blue (Sigma-Aldrich), and oil red (Sigma-Aldrich) was confirmed after 21 days of culture as described elsewhere [32,48]. Thus, the isolated cells constitute a pool of human adipose-derived multipotent mesenchymal stromal/stem cells (hAMMSCs).

### 4.5. In Vitro Cell Culturing and Alizarin Red Staining

To study the cytotoxic effect of the sample (10 mg/L), a suspension of hAMMSC was prepared at a concentration of 5 × 10^4^ viable cells/mL in 1.5 mL of culture medium (90% aMEM medium (Gibco Life Technologies; Grand Island, New York, NY, USA), 10% fetal bovine serum (Sigma-Aldrich, St. Louis, MO, USA), 50 mg/L gentamicin (Invitrogen, Carlsbad, CA, USA), a sterile solution of l-glutamine added to the final concentration of 280 mg/L (Sigma-Aldrich, St. Louis, Missouri MO, USA). The cells were cultured in a 24-well flat-bottomed plate (Orange Scientific, Braine-l’Alleud, Belgium) for 21 days. The medium was replaced with a fresh medium every 3–4 days. Tested CaChA was added initially and each time while the medium was replaced. Cell viability was determined with a Countess^TM^ Automated Cell Counter (Invitrogen, Carlsbad, CA, USA) using 0.4% trypan blue solution (Invitrogen, Carlsbad, CA, USA) as described previously [32]. The numbers of viable (unstained) cells were determined and the percentages from the control culture (without test compound) were calculated.

To determine the influence of CaChA on the differentiation of hAMMSCs into osteoblasts, another three wells of the flat-bottom plate for each test group were fixed with 10% formalin for 1 h, washed with phosphate buffer, and stained with 2% alizarin red S (Sigma-Aldrich, St. Louis, MO, USA) to visualize mineralization of osteoblasts and surrounded extracellular matrix. Staining was performed as recommended by the manufacturer. Digital images of the hAMMSC culture were obtained and assessed with a Zeiss Axio Observer A1 microscope (Carl Zeiss Microscopy, LLC, Thornwood, NY, USA) using ZEN 2012 software (Carl Zeiss Microscopy, LLC). The average area of alizarin red S staining (in mm^2^), total area (average area × number of stained sites), and an optical density (OD) of the mineralizing sites were calculated in each well via quantitative computer histomorphometry with the help of ImageJ v. 1.43 software (National Institutes of Health, Bethesda, Montgomery MD, USA; http://www.rsb.info.nih.gov/ij (accessed on 28 June 2012)) and Adobe Photoshop version 13.1.2 Software as described earlier [32,51]. The percentages of indices with the corresponding values of control culture (without test compound) were calculated.

### 4.6. Ectopic Osteogenesis Test to Study In Situ Osteogenic Activity of Calcium Chelidonate

To study CaChA ability to promote osteoinduction in situ, well-known ectopic bone formation test was used in vivo. Subcutaneous in situ implantation is a simple and widely relevant location [52]. In vivo investigation was carried out on 15 Balb/c male mice in compliance with the principles for the humane treatment of laboratory animals [53]. The animal experiment was approved by the Local Ethics Committee of Immanuel Kant Baltic Federal University (Permission no. 7 from 9 December 2015). In this study, ten animals were used for subcutaneous implantation, while five animals served as bone marrow donors.

There were a few reports stating that CaP scaffolds without cells had not new bone formation after subcutaneous implantation [48,54]. Hence, well–known variant of ectopic test with syngeneic bone marrow showed reproducible (75–100%) bone/marrow remodeling on micro-arc CaP coating implanted subcutaneously [42,55].

For implantation, 10 titanium plates ((10 × 10 × 1) mm^3^) were prepared from pure titanium VT1-0. Titanium plates were coated with calcium phosphate (CaP) by microarc oxidation (MAO) using the Microarc 3.0 system (ISPMS SB RAS, Tomsk, Russia) as described earlier [48]. Before the experiment, the plates were sterilized by dry heat in the Binder FD53 oven (Binder GmbH, Tuttlingen, Germany) at 453 K for 1 h.

The CaP-coated Ti substrates were preliminarily incubated in vitro for 45 min at 37 °C in a culture medium consisting of 95% DMEM medium (Sigma-Aldrich, St. Louis, MO, USA) and 5% fetal bovine serum (Sigma-Aldrich, St. Louis, MO, USA) to adhere the syngeneic bone marrow. Bone marrow columns were isolated from mouse femurs by flushing the culture medium with a syringe. Bone marrow serves as a mesenchymal stromal/stem cell source for new bone formation (i.e., osteoinduction). Each sample covered by bone marrow column (5–8 mm^2^ marrow per sample) was photographed (PowerShot A 630, Canon, Japan) and implanted under etherization into the animal venter lateral subcutaneous pocket and the wound was sutured as described previously [55].

CaChA water solution was administered daily at a dose of 10 mg/kg of mouse body mass (0.5 mL per animal) using a peroral pathfinder for 35 days after subcutaneous implantation of CaP-coated Ti samples. Control mice were treated with a pure solvent in the corresponding volume.

After 45 days, the animals were removed from the experiment with an overdose of carbon dioxide. The implants were explanted, fixed in formalin, and decalcified. Tissue lamellae formed from the bone marrow were removed from the surface of the implants, dehydrated and immersed in paraffin to obtain histological sections. Thin (8 μm) cross sections were dewaxed and stained with hematoxylin and eosin, as described elsewhere [55]. To estimate the histological composition of tissues grown on the implants’ surface, stained tissue sections of the lamellae were examined microscopically (Axioskop 40, Carl Zeiss, Germany) and their digital images (Power Shot A 630, Canon, Japan; 14 megapixels resolution) were done. Bone tissue with or without bone marrow was considered in histological sections of lamellae as a positive result of the ectopic osteogenesis test.

The incidences of the lamella and ectopic bone formation on the surface of the implants were determined. The median areas (mm^2^) covered by the bone marrow before implantation and the grown tissues were calculated via quantitative computer histomorphometry using Image J 1.43 software (National Institutes of Health, Bethesda, Montgomery MD, USA; http://www.rsb.info.nih.gov/ij (accessed on 28 June 2012)).

### 4.7. Statistical Analysis

Statistical analysis was carried out via the Statistica 13.3 software. The data are shown as the median (Me), 25% quartile (Q1) and 75% quartile (Q3). The normality of the distribution was defined by the Kolmogorov-Smirnov test. Due to the non-normal distribution, a nonparametric Mann-Whitney test was used to evaluate the significant differences between the groups. Statistically significant differences were considered at the value of *p* < 0.05.

## 5. Conclusions

Calcium chelidonate [Ca(ChA)(H_2_O)_3_]_n_ is extracted from the plant and the resulting semisynthetic has an identical structure to that confirmed by X-ray analysis. The asymmetric unit of [Ca(ChA)(H_2_O)_3_]_n_ includes chelidonic acid coordinated to three water ligands. The atoms of acid and water from each asymmetric unit are also coordinated with those of another calcium, forming an infinite linear complex. The intracomplex hydrogen bonds additionally stabilize linear species, which are parallel to the axes. In turn, the linear species are packed into the 3D structure through mutual intercomplex hydrogen bonds.

The enhanced ECM mineralization in vitro and bone tissue regeneration in situ were observed while a water solution of calcium chelidonate at a dose of 10 mg/kg was used. Thus, the test substance promotes hAMMSCs, as well as mouse MSCs to differentiate into osteoblasts in vitro and in vivo, respectively. The test substance is non-toxic and can stimulate OI process.

## Figures and Tables

**Table 1:** Selected geometrical parameters of [Ca(ChA)(H_2_O)_3_]_n_.

Bonds, (Å)			
Ca1–O1	2.630(2)	Ca1–O10	2.459(2)
Ca1–O2	2.480(2)	Ca1–O11	2.419(3)
Ca1–O3	2.397(3)	Ca1–O2_a	2.483(3)
Ca1–O4	2.330(3)	Ca1–O10_a	2.555(2)
**Angles, (°)**			
O1–Ca1–O2	127.95(8)	O2–Ca1–O2_a	115.13(8)
O1–Ca1–O3	79.59(9)	O2–Ca1–O10_a	76.73(7)
O1–Ca1–O4	131.19(8)	O3–Ca1–O4	77.93(10)
O1–Ca1–O10	61.84(7)	O3–Ca1–O10	77.64(8)
O1–Ca1–O11	61.18(7)	O3–Ca1–O11	3.38(9)
O1–Ca1–O2_a	71.63(7)	O3–Ca1–O2_a	150.01(9)
O1–Ca1–O10_a	137.97(6)	O3–Ca1–O10_a	142.42(8)
O2–Ca1–O3	76.38(9)	O4–Ca1–O10	148.79(9)
O2–Ca1–O4	87.34(9)	O4–Ca1–O11	77.56(9)
O2–Ca1–O10	68.22(7)	O4–Ca1–O2_a	128.01(9)
O2–Ca1–O11	163.33(8)	O4–Ca1–O10_a	75.06(8)

**Table 2:** Parameters of hydrogen bonds for [Ca(ChA)(H_2_O)_3_]_n_.

Hydrogen Bond	O/C–H, (Å)	H…O, (Å)	O/C…A, (Å)	O/C–H…A, (°)
	intra–complex			
O2–H2A…O12	0.83(3)	1.81(4)	2.602(3)	160(4)
O3–H3A…O11	0.84(3)	2.20(3)	3.024(4)	166(4)
	inter–complex			
O2–H2B…O9	0.84(4)	1.98(4)	2.789(3)	163(3)
O3–H3B…O4	0.84(3)	2.35(4)	3.043(4)	139(5)
O3–H3B…O11	0.84(3)	2.38(4)	3.094(4)	142(5)
O4–H4A…O9	0.84(3)	1.89(3)	2.721(4)	170(3)
O4–H4B…O13	0.85(4)	1.87(4)	2.686(4)	162(4)
C5–H5…O12	0.93	2.42	3.327(4)	166

**Table 3:** Effect of CaChA in vivo administration on the tissues growing subcutaneously on CaP-coated Ti substrates after 45 days of the ectopic test in mice, Me (Q1; Q3).

The Groups Studied, *n* = 5	The Incidence of Tissue Lamella Growth on CaP Surface	The Incidence of Ectopic Bone Formation in Lamella	Bone Marrow Column Seeded In Vitro on CaP Coating (Initial Levels before Implantation)	Tissue Lamella Properties In Situ (after Implantation)				
	%	%	Area, mm^2^	Area of Tissue Lamellae, mm^2^	Number of Calculated Cross Sections Per Lamella	Part of Cross Sections with bone, %	Area of Newborn Bone Per Cross Section, mm^2^	Histological Composition
CaP-coated samples under the skin + water solvent per os (control)	80 (4/5)	75 (3/4)	7.65 (6.63–8.17)	23.65 (18.40–31.90)	141 (36–167)	78 (31–100)	0.21 (0.13–0.24)	Bone with marrow (Figure 4a–c) in 3 cases; connective and adipose tissues (not shown) in 1 case
CaP-coated samples under the skin + CaChA per os, 10 mg/kg	100	100	6.27 (5.94–7.36)	57.40 * (42.00–60.40)	89 (81–132)	100 (96–100)	0.20 (0.07–0.23)	Bone tissue with bone marrow (Figure 4d–h) in 5 cases

*n*—number of CaP-coated Ti samples studied in each group; * statistical differences (*p* < 0.05) with control according to a Mann–Whitney test.

## Data Availability

The data presented in this study are available in article and supplementary material.

## Supplementary Materials

The following are available online at https://www.mdpi.com/article/10.3390/ph14060579/s1, Table S1: Crystallographic parameters and details of experiment solution and refinement for semi-synthetic and natural forms of [Ca(ChA)(H_2_O)_3_]_n_.
