# Supplementary material for: Calcium Chelidonate: Semi-Synthesis, Crystallography, and Osteoinductive Activity In Vitro and In Vivo
Source: Pharmaceuticals (Basel). 2021 Jun 17;14(6):579. doi: 10.3390/ph14060579 (PMC8235635; doi:10.3390/ph14060579)
Supplement: Supplementary file 1 [file pharmaceuticals-14-00579-s001.zip › pharmaceuticals-1248189-supplementary.pdf]

Supplementary

Calcium Chelidonate: Semi-synthesis, Crystallography, and Osteoinductive Activity In Vitro and In Vivo

Elena Avdeeva[1]*, Ekaterina Porokhova[2], Igor Khlusov[2,3], Tatyana Rybalova[4,6], Elvira Shults[5,6], Larisa Litvinova[7], Valeria Shupletsova[7], Olga Khaziakhmatova[7], Irina Sukhodolo[2], and Mikhail Belousov[1,3]

[1] Department of Pharmaceutical Analysis, Siberian State Medical University, Tomsk, 634050 ,Russia; mvb63@mail.ru (M.B)
[2] Department of Morphology and General Pathology, Siberian State Medical University, Tomsk, 634050, Russia; porohova_e@mail.ru (E.P.); staranie@mail.ru (I.S.); khlusov63@mail.ru (I.K.)
[3] Research School of Chemistry & Applied Biomedical Sciences, Tomsk Polytechnic University, Tomsk, 634050, Russia
[4] Center of Spectral Investigations, Novosibirsk Institute of Organic Chemistry, Siberian Branch, Novosibirsk, 630090, Russia; rybalova@nioch.nsc.ru (T.R)
[5] Laboratory of Medicinal Chemistry, Novosibirsk Institute of Organic Chemistry, Siberian Branch, Novosibirsk, 630090, Russia; schultz@nioch.nsc.ru (E.S.)
[6] Novosibirsk State University, 2 Pirogova St., Novosibirsk 630090, Russia
[7] Basic Laboratory of Immunology and Cell Biotechnology, Immanuel Kant Baltic Federal University, Kaliningrad, 236041, Russia; larisalitvinova@yandex.ru (L.L.); vshupletsova@mail.ru (V.S.); hazik36@mail.ru (O.K.)
* Correspondence: elenaavdeev@yandex.ru; Tel.: +7-983-344-7381 (E.A.)

Table S1. Crystallographic parameters and details of experiment solution and refinement for semi-synthetic (**II**) and natural (**I**) forms of $[Ca(ChA)(H_2O)_3]_n$

	avd2_synt (**II**)	avd2_nat (**I**)
Empirical formula	$C_7H_8CaO_9$	$C_7H_8CaO_9$
Formula weight	276.21	276.21
Crystal system	Orthorhombic	Orthorhombic
Space group	Pna2(1)	Pna2(1)
Unit cell dimensions	a = 8.380(2) A alpha = 90 deg.	a = 8.419(13) A alpha = 90 deg.
	b = 19.702(4) A beta = 90 deg	b = 19.82(3) A beta = 90 deg.
	c = 6.1653(14) A gamma = 90 de	c = 6.207(8) A gamma = 90 deg.
Volume	1017.9(4) A^3	1036(3) A^3
Z, Calculated density	4, 1.802 Mg/m^3	4, 1.772 Mg/m^3
Absorption coefficient	0.655 mm^{-1}	0.644 mm^{-1}
F(000)	568	568
Crystal size	0.30 x 0.09 x 0.005 mm	0.30 x 0.09 x 0.01 mm
Theta range for data collection	2.64 to 25.93 deg.	2.63 to 24.79 deg.

Limiting indices	-9<=h<=10, -24<=k<=24, -7<=l<=7	-8<=h<=9, -23<=k<=20, -7<=l<=6
Reflections collected / unique	9349 / 1966 [R(int) = 0.0487]	4896 / 1608 [R(int) = 0.1379]
Completeness to theta = 24.79	99.1 %	99.1 %
Absorption correction	Semi-empirical from equivalents	Semi-empirical from equivalents
Max. and min. transmission	0.8620 and 0.7714	0.8620 and 0.5294
Refinement method	Full-matrix least-squares on F^2	Full-matrix least-squares on F^2
Data / restraints / parameters	1966 / 7 / 179	1608 / 11 / 166
Goodness-of-fit on F^2	1.065	1.000
Final R indices [I>2sigma(I)]	R1 = 0.0347, wR2 = 0.0765	R1 = 0.0721, wR2 = 0.1508
R indices (all data)	R1 = 0.0446, wR2 = 0.0801	R1 = 0.1230, wR2 = 0.1716
Absolute structure parameter	0.12(4)	0.00(12)
Largest diff. peak and hole	0.322 and -0.220 e.A^{-3}	0.716 and -0.603 e.A^{-3}
